# Tissue Engineering of Oral Mucosa and Salivary Gland: Disease Modeling and Clinical Applications

**DOI:** 10.3390/mi11121066

**Published:** 2020-11-30

**Authors:** Akram Abdo Almansoori, Bongju Kim, Jong-Ho Lee, Simon D. Tran

**Affiliations:** 1 Department of Oral and Maxillofacial Surgery, School of Dentistry, Seoul National University, Seoul 03080, Korea; dentist22@gmail.com (A.A.A.); leejongh@snu.ac.kr (J.-H.L.); 2 Clinical Translational Research Center for Dental Science, Seoul National University Dental Hospital, Seoul 03080, Korea; bjkim016@gmail.com; 3 McGill Craniofacial Tissue Engineering and Stem Cells Laboratory, Faculty of Dentistry, McGill University, 3640 University Street, Montreal, QC H3A 0C7, Canada

**Keywords:** microengineering, oral mucosa, salivary gland, 3D cell culture

## Abstract

Oral mucosa and salivary gland are composed of complex and dynamic networks of extracellular matrix, multiple cell types, vasculature, and various biochemical agents. Two-dimensional (2D) cell culture is commonly used in testing new drugs and experimental therapies. However, 2D cell culture cannot fully replicate the architecture, physiological, and pathological microenvironment of living human oral mucosa and salivary glands. Recent microengineering techniques offer state of the science cell culture models that can recapitulate human organ structures and functions. This narrative review describes emerging in vitro models of oral and salivary gland tissue such as 3D cell culture models, spheroid and organoid models, tissue-on-a-chip, and functional decellularized scaffolds. Clinical applications of these models are also discussed in this review.

## 1. Introduction

Oral mucosa and salivary glands are composed of complex and dynamic networks of extracellular matrix, multiple cell types and neuro-vasucaluar networks. The oral mucosa is composed of three layers: superficial layer epithelium, basement membrane, and a core of lamina propria (connective tissue). The epithelium is made of stratified squamous epithelium and the lamina propria consists of collagen and fibroblasts. The basement membrane is a thin layer composed of collagen and laminin [1]. Salivary glands are exocrine structures composed mainly of serous acini, ductal, and myoepithelial cells that produce and secrete saliva [2]. Ongoing research is trying to reproduce the oral mucosal and salivary glands in term of their unique structures and physiological interactions [3,4,5]. Two-dimensional (2D) cell culture is commonly used in testing new drugs and experimental therapies. However, 2D cell culture cannot fully replicate the architecture, physiological, and pathological microenvironment of living human oral mucosa and salivary glands [6,7,8]. Animal models are also being used extensively, but they are surrounded by many ethical and technical issues [9,10,11,12].

Recent microengineering techniques offer state of the science cell culture models that can recapitulate human organ structures and functions. They are offering minimal functional units that are usually made of human cells in 3D architecture and perfused with fluid simulating the body circulation. It allows a direct assessment of cellular biochemical and metabolic activities. This narrative review describes emerging in vitro models of oral and salivary gland tissue such as 3D cell culture models, spheroid, and organoid models, tissue-on-a-chip, and functional decellularized scaffolds. Clinical applications of these models are also discussed in this review.

## 2. Three-Dimensional Cell Culture Modalities

### 2.1. Spheroid

There are several 3D culture techniques that are aiming at the formation of cell aggregates, which are called spheroids. Examples of these techniques include spontaneous cell aggregation, hanging drop, and rotating culture vessels [13,14]. These models allow cell–cell interactions, cell polarity and differentiation, and recapitulating the external cellular matrix properties. However, these models lack control in spheroid sizes, predictivity, and mechanical stimuli. Usually as the spheroid is getting larger in size, cell necrosis occurs in the core of the cell aggregates [15,16].

### 2.2. Organoid

Organoids are produced by seeding cells into basement membrane substrate like hydrogel matrix [17]. The organoid structures have better size uniformity, cell polarization, and cell–cell interactions. The main limitations are the presence of xenogeneic materials, uncontrollable degradation of the scaffolds, and a lack of mechanical stimulation [14,16,18].

### 2.3. 3D Microfluidic Cell Culture System

These are microfluidic devices housing spheroid/organoid and microfluidic channels. They are more physiologically relevant in vitro models. The microfluidic channels perfuse the cells with fluid containing nutrients and subjecting them to mechanical stress. The system allows co-culture of multiple cellular layers and cell–cell interactions. The chip is produced generally by photolithography. Microsensors are built-in to detect variable electrical, physical and chemical stimuli. However, the chips are complicated to fabricate and operate for cell culture, and are not yet standardized [16,19,20].

### 2.4. Functional Decellularized Scaffolds

In the field of solid organ transplantation, various studies have successfully decellularized and regenerated different organs like the lung, liver, and kidney [21]. With complete removal of the donor cellular components, the immune rejection is prevented or reduced to a minimum [22,23,24,25]. Various decellularizing agents have been used such as alkaline compounds, acids, alcohols, detergents, and enzymes [21]. The main goal of this approach is to remove the cells and preserve the ECM components like collagen, glycosaminoglycans, laminin, and growth factors [26]. The production of organ scaffolds lacking cellular content but have functional vascular and interstitial compartments may serve as a valuable physiological model for investigating organ development and pathogenesis [27].

## 3. Oral Mucosa 3D Culture Models

Oral mucosa harbors diverse oral flora microorganisms and is subjected to various dental materials. Several oral mucosa models have been created to evaluate the biomaterials biocompatibility [28,29]. 3D cell culture is a source for producing abundant functional oral epithelial cells to be used in tissue engineering compared to the monolayer epithelia cells which tend to change their phenotype to epithelial-mesenchymal or mesenchymal [30]. Several 3D models have been employed like the scaffold-based culture, hanging drop, suspension culture, and oral mucosa-on-a-chip.

### 3.1. Scaffold Based Culture

Scaffold based culture models have been developed for testing the effect of dental restorative materials and bacteria on oral mucosa cells. The cells that are usually used for the oral mucosa 3D culture model are oral fibroblasts and keratinocytes. Multiphoton microscopy proved to be useful for noninvasive and real time imaging of 3D cell culture containing oral fibroblasts and keratinocytes [31]. Collagen matrix provided a proper support for the adhesion, proliferation, and phenotypic expression of keratinocytes and fibroblasts [32]. In a reported study, keratinocytes and fibroblasts were co-cultured over a collagen matrix and subjected to 5-fluorouracil (5-FU) to produce a chemotherapy induced oral mucositis model. The histopathological changes induced by 5-FU in this model were close to those produced in vivo. These changes included induction of cells apoptosis and stimulation of pro-inflammatory cytokines [33]. Another collagen-based oral mucosa model was used to assess the interaction with bacterial and fungal microorganisms. *Candida albicans* caused significant release of lactate dehydrogenase that may facilitate the invasion of the tissue by *Staphylococcus aureus* [34].

Using Matrigel™-ECM culture system, the effect of triethyleneglycol dimethacrylate (TEGDMA) was assessed against mouse gingival fibroblasts and dental papilla mesenchymal cells (DPMCs) organoids. TEGDMA was found to interrupt the formation of DPMCs organoids at low dose in contrast to the monolayer culture cell model [35]. The culture model plays a significant role in the functionality and size of cultured cells-derived structures. In one study, small extracellular vesicles (SEVs) secreted by 3D cultured oral mucosa lamina propria-progenitor cells were abundant and caused a decrease in the proliferation of the co-cultured fibroblasts. On the other hand, SEVs derived from 2D culture cells were fewer and increased the proliferation of fibroblasts. Such findings might be useful for wound healing applications [36]. A two-stage culture system, non-adhesive agarose plates and hybrid matrix (poly(lactide-coglycolide) (PLGA) and collagen), was valuable for the formation of a multilayered epithelial lining from oral mucosa epithelial cells. The epithelial cells maintained their phenotypes and expressed polarity and intercellular junctions [30]. 3D cell culture to study oral cancer is a valuable system too. Oral squamous cancer cells have shown different expression of N-cadherin and E-cadherin in 3D multicellular spheroids when compared to 2D culture cells which suggests that cell–cell expression is dynamic and changes depend on the cellular needs [37].

Several studies produced models to assess the interaction between the oral epithelium and dental implant surface. Using a reconstructed human gingiva model, the epithelial attachment to the implant surface was evaluated. The model showed formation of gingival margin, junctional epithelium, and sulcus, with variable expression of keratin 4 and 19 along these sites [38]. In another study, an in vitro oral mucosa model was created to evaluate the effect of oral commensal and pathologic bacteria on a peri-implant mucosa. The model composed of oral mucosa construct, implant material, and oral biofilm (*Streptococcus oralis or Aggregatibacter actinomycetemcomitans*). The *S. oralis* led to a slight tissue loosening which might facilitate a fast transmigration of polymorphonuclear neutrophils to tissue-biofilm interface and build a barrier against microbial invasion. However, the *A. actinomycetemcomitans* decreased the inflammatory response that may facilitate the bacteria colonization and invasion of the tissue later [39]. The early interaction between the biofilm and peri-implant mucosa was investigated too. During the first 24 h, there were no significant findings except a weak pro-inflammatory response. After 48 h, the epithelial barrier was disrupted, and the mucosa was detached from the implant along with enhanced pro-inflammatory response through secretion of CCL20, IL-1β, and TNF-α [40].

### 3.2. Suspension Culture

The hanging drop or suspension culture system are also useful for the formation of oral mucosa-derived cells spheroids. The formed spheroids have been used to study inflammatory and infection disease models. In one study, the spheroid model consisting of keratinocyte-fibroblasts was infected with *Porphyromonas gingivalis*. The bacteria increased cell death, inflammatory markers, and bypassed the epithelia barrier into the fibroblastic core to induce the disorganization of the spheroid [41]. In another study, spheroid-derived gingival mesenchymal stem cells expressed high levels of reactive oxygen species, hypoxia-inducible factor (HIF)-1 and -2 α, and manganese superoxide dismutase, and promoted regeneration of chemotherapy-induced oral mucositis in a murine model [42].

### 3.3. Oral Mucosa-on-A-Chip

Recently, oral mucosa-on-a-chip has been developed to evaluate the response of epithelial and subepithelial layers to dental restorative materials, drugs, and bacteria. It was fabricated by embedding a submucosal layer of fibroblasts in collagen in a central channel with interconnecting pores and coating a keratinocyte layer to the collagen exposed in the pores. Exposure of this oral construct to dental monomer has lowered mucosal cell viability while the oral bacteria *Streptococcus mutans* lowered the transepithelial electrical resistance [28]. Using another microfluidic-based 3D co-culture device, carcinoma-associated fibroblasts proved to have a significant role in cancer invasion though promoting salivary gland adenoid cystic carcinoma (ACC) cell invasion of tumor spheroid [43] (Table 1).

## 4. Salivary Gland 3D Culture Models

### 4.1. Scaffold Based Culture

There are several 3D culture models that have been tested for salivary gland cells. Scaffold-based culture, hanging drop, non-adherent surface, and bioprinting all have demonstrated variable outcomes (Table 1). Scaffold-based hydrogels are made from animal tissue extracted proteins like collagen gel, fibrin gel, and Matrigel^®^ [44]. The salivary gland cells are seeded in the gel and divide to form spheroids that can differentiate into acinar-like structures and express tight junction proteins like occludin and water channel protein like AQP5 [44,45,46]. Fibrous scaffolds have also supported the development of salivary spheroid. They are characterized by their in vivo-like topography and porous nature that support nutrient delivery and waste removal [18]. These materials include natural polymers like chitosan, and synthetic polymers like polyethylene glycol and poly(lactic-*co*-glycolic acid). Scaffold-based matrices have supported salivary cell aggregation, polarization, and salisphere formation. However, their main limitations are the presence of xenogeneic substances and uncontrolled degradation rate [18,44].

Biomechanical factors play important roles in the development of the salivary spheroid. A study reported that the addition of FGF-10 into spheroid culture has resulted in an increased expression of acinar markers and more sensitivity to neurotransmitters [47]. In the same study, combining spheroids and the salivary mesenchyme mimic the native salivary gland properties in terms of morphology and saliva secretion when transplanted into mice [47]. However, because the mesenchyme used in the study was an animal-derived structure, this method is not yet applicable for clinical practice [47]. Hydrogel-formed organoid has shown increased granule production and amylase staining [48]. Activin receptor-like kinase (Alk) inhibitors have facilitated the formation of salivary organoids within a Matrigel culture-based system [49]. The same model has offered a simulation for salivary gland disease as the long term TNF-α stimulation decreased AQP5 expression as in sialadenitis [49]. Branching morphogenesis of culture epithelial cells in a Laminin-111 coated hydrogel-based culutre system has been induced through the application of the neurotrophic factor, neurturin [50]. One of the main drawbacks of bioscaffolds is the presence of xenogenic material. In an effort to find suitable hydrogel-based culture systems as alternatives to Matrigel for clinical applications, human fibronectin and human basement membrane extracts have been successfully used for the culture of human salivary gland cells and induced their differentiation into acinar-like structures [51,52]. In another interesting study, a cost-effective and native extracellular matrix was recycled by collecting the residual connective tissue that remained after cells isolation through salivary tissue mincing and enzymatic digestion. It was called the natural ExtraCellular Matrix scaffold (nECMsc). The nECMsc looked like a gelatinous mass and showed comparable morphology, proteins composition, and epithelial cell seeding efficiency when compared to the native salivary gland tissue. The seeded epithelial cells attached, proliferated and remained viable after 4–8 days of culture period [26].

Salivary gland hypofunction following pathological conditions or head and neck radiotherapy usually leads to tremendous impairment of oral health, speech, and swallowing [53,54]. The 3D culture techniques for salivary cell culture are aimed at producing optimal and functional salivary cells and organoids to be used to repair or replace damaged glands. A radiation-damaged salivary tissue model was proposed [55]. Salivary cells were obtained from human parotid glands and cultured on 2D plastic plates and 3D Matrigel based culture systems. The 3D formed spheroids were exposed to irradiation at 10 and 20 Gy. The spheroid model allowed the measurement of functional alteration subsequent to irradiation better than the 2D monolayer model, such as the measurements of loss of α-amylase secretion in response to cholinergic or β-adrenergic agonists [55]. For salivary gland cancer, poorly differentiated human mucoepidermoid carcinoma cells were 3D cultured in sodium alginate gel microcapsules and compared to 2D culture cells. The 3D culture cells exhibited a stronger proliferation and secretion of VEGF-A and bFGF than 2D culture cells [56] (Table 2).

### 4.2. Suspension Culture

Non-adherent surfaces and suspension culture systems are offering simple scaffold-free methods for the formation of spheroids. Nevertheless, the spheroids are slow to form, non-uniform, and of uncontrollable sizes [18]. Partial digestion of the salivary gland tissue and culturing the cells in a serum-free scalable suspension culture has resulted in functional salivary spheroids called salivary functional units. It was proposed that partial tissue digestion allowed the cells to maintain their native extracellular matrix (ECM) [57]. The formed salivary spheroids expressed cell polarization, acinar cell markers, and tight junction proteins. The absence of animal-derived serum makes this protocol amenable for clinical applications. The salivary spheroids increased in size by both cell proliferation and cell aggregation during the 5–10 days cell culture period. From there, cell apoptosis started due to the uncontrollable increase in spheroid size and a limitation in nutrients and oxygen diffusion rate. Culturing of these salivary functional units in 3D ECM-derived matrix may maintain their structure integrity over 10 days [26,57].

### 4.3. Microwells Culture

Recently, the formation of more uniformed acinar-like spheroids in microwells of hydrogel micropatterning and nanofibrous scaffold was reported [58]. These microwell culture systems have shown a higher expression of salivary acinar, ductal, and tight junction markers when compared to 2D and 3D culture systems like Matrigel or suspension cultures [59]. The formed spheroids showed higher α-amylase secretion and intracellular calcium levels in response to adrenergic or cholinergic agonists [59]. This system is also offering a niche independent culture system within serum-free culture medium for the formation of uniform spheroids [58,59].

### 4.4. Bioprinting Culture

Bioprinting technologies have allowed labelling of the cells with magnetic nanoparticles to freely assemble them into more uniformly sized spheroids faster. This provides a consistent method to form salivary organoids [60]. However, the main drawbacks are biocompatibility and requirement for specialized equipment [60,61]. In a recent study, human salivary acinar cells and primary fibroblasts were cultured inside a gelled egg yolk plasma (GEYP) within a 3D-Cryo insert wells for 14 days. GEYP has the advantages of being cost-effective and abundant. The cell culture growth was maintained using either culture media or egg white. In addition, GEYP was successfully 3D printed with controlled geometrics. As a simulation for the epithelial-mesenchymal interface, GEYP bio-ink with fluorescence cells was manually extruded inside the Cryo-well insert and showed reproducible cell positioning [61].

### 4.5. Decellularized Scaffold

To re-create the microenvironment of native salivary gland, decellularized salivary gland scaffolds have been used for 3D culturing of the salivary gland cells [26]. One method mounted the scaffold on a rotary cell culture system and cells were seeded through the gland duct. Cells showed adhesion to the scaffold and expressed acinar differentiation markers [62]. In the reported study, the authors have also stressed the need for orthotopic transplantation of the bioengineered salivary glands using a large animal model like miniature pig [62]. A decellularized extracellular matrix-hydrogeld culture system has been produced too. It represented a functional orthotropic bioscaffold that supported the survival of salivary gland stem/progenitor cells and their acinar differentiation [63] (Table 2). 

## 5. Conclusions

Oral mucosa and salivary glands are composed of unique structures. In vitro culture systems are rapidly evolving to reproduce their native histological structures and biochemical activities. These in vitro culture systems include scaffold-based matrix, suspension culture, microwell culture, bio-printing, decellularized extracellular matrix, and tissue on a chip. They offer a promising way to produce functional oral and salivary cells/spheroids that can be used for tissue engineering and testing the interaction with various drugs, dental restorative materials, and microorganisms.

So far, oral mucosa-derived spheroids have been used for many oral disease models. Specific gingival spheroids were also produced and used for the evaluation of gingiva-bacteria interaction. A formed multilayered epithelial lining was also proposed for oral mucosa defect reconstruction. Recently, oral mucosa microfluidic-based device fabrication has significantly advanced the assessment of oral mucosa cells and external agents through in vivo-mimicking microenvironment. Despite this advancement, there are still some limitations in the quality of the formed oral mucosa-derived structures such as maintaining the oral epithelial cell phenotypes and the creation of a proper oral epithelium-lamina propria interface.

On the other hand, salivary 3D culture models have successfully produced functional salivary sphreroids/cells that showed promising results in the restoration of irradiation-damaged glands. The addition of FGF-10, Neurturin, or Activin receptor-like kinase (Alk) inhibitors to the salivary 3D cell culture had promoted the development of branching morphogenesis and salivary organoids. Salivary gland disease model (sialadenitis) was also proposed using a scaffold-based matrix. However, one of the major limitations of the scaffold-based matrix is the presence of xenogeneic material. Therefore, Human Fibronectin, Human Basement Membrane Extract and Natural extracellular matrix scaffolds (NEMsc) were successfully tested as alternatives for clinical applications. Furthermore, the partial digestion of human submandibular tissue and culture of cells within suspension culture systems allowed the cells to attach to their native extracellular matrix components and produced salivary functional units. Despite this advancement in salivary spheroid formation, some limitations needed also to be addressed. These limitations include the slow growth rate and short-term viability of the salivary cells and the uncontrollable size of the salivary spheroids. The produce of uniformed size spheroids was achieved by the introducing of microwells culture systems. Furthermore, bio-printing models such as magnetic 3D levitation and GEYP bio-printing models provided the chance for consistent organoid formation. The formation of fully functional salivary organoids and integrating them in microfluidic-based devices is still in an early stage of development. Such developments will allow the assessment of various drugs including radioprotective agents. Interestingly, decellularized salivary gland scaffold with preserved blood vessels is providing a native perfused scaffold as an alternative promising model to microfluidic-based devices.

## Figures and Tables

**Table 1:** Oral mucosa 3D culture models.

System	Method	Cell Type	Observations	References
Scaffold-based	Collagen matrix	Gingival fibroblasts & oral keratinocytes	Suitable substrate for oral keratinocytes adhesion, proliferation, and phenotypic expression	[32]
Scaffold-based	Collagen matrix	Human oral keratinocytes cell line & fibroblasts cell line	In vitro chemotherapy-induced mucositis model. Oral commensals may play a role in pathogenesis of oral mucositis.	[33]
Scaffold-based	Cellularised, collagen gel	Human keratinocytes & gingival fibroblasts	Reconstituted oral mucosa tissue Infection model for *Candida albicans* and *Staphylococcus aureus* *C. albicans* caused high release of lactate dehydrogenase that may facilitate the damaging effect of *S. aureus*.	[34]
Scaffold-based	Matrigel™-ECM	Mouse gingival fibroblasts & mouse dental papilla mesenchymal cells	Low TEGDMA doses altered and decreased the thickness of the dental papilla mesenchymal cells organoid.	[35]
Scaffold-based	Type I collagen matrix	Human oral mucosa lamina propria-progenitor cells & skin fibroblasts	SEVs produced in 3D cell culture significantly reduced the proliferation of skin fibroblasts. SEVs produced in 3D cell culture were of greater amount.	[36]
Scaffold-based	Agarose-coated plates, serum-free media	SCC9β6KDFyn cell line	Differential expression of N-cadherin and E-cadherin by oral SCC cells. “Cadherin switching” dynamic and depends on the cellular needs.	[37]
Scaffold-based	Non-adhesive agarose plates Hybrid matrix of PLGA and collagen.	Oral mucosa buccal epithelial cells	Multilayered epithelial lining formation.	[30]
Scaffold-based	Collagen gel	Human gingival fibroblasts & oral keratinocytes	In vitro peri-implant mucosa-biofilm model. *S. oralis* induced a protective inflammatory response. *A. actinomycetemcomitans* reduced the inflammatory response.	[39]
Scaffold-based	Collagen hydrogel	Immortalized human gingiva keratinocytes and fibroblast cell line	Assessed the soft tissue attachment to two different abutment surfaces. Formation of gingival margin, sulcular, and junctional epithelium. The sulcus depth and junctional epithelial length were similar to pre-clinical and clinical lengths.	[38]
Scaffold-based	Collagen hydrogel	Human gingival fibroblasts & Immortalised human oral keratinocytes	24 h commensal biofilm exposure to the peri-implant mucosa: weak pro-inflammatory response. 48 h biofilm exposure: epithelial barrier disrupted and the mucosa was detached from the implant.	[40]
Hanging drop	Hanging drop	Human gingival epithelial cells & human oral fibroblasts	Gingival spheroid model. *P. gingivalis* increase cell death and inflammatory markers. *P. gingivalis* bypassed the epithelial barrier to reach the fibroblastic core.	[41]
Suspension Culture	Suspension culture	Human Gingiva-Derived Mesenchymal Stem Cells	Spheroid gingival stem cells expressed stem cell phenotype markers. Promoting the regeneration of chemotherapy-induced oral mucositis.	[42]
Oral mucosa–on-a-chip	Microfluidic-based oral mucosa model	Keratinocyte, fibroblast, and collagen	Useful for assessment of oral mucosal interactions with bacteria and biomaterials. Exposure to dental monomer lowered mucosal cell viability. Exposure to the oral bacteria *Streptococcus mutans* lowered the transepithelial electrical resistance.	[28]
Oral mucosa–on-a-chip	Microfluidic-based oral mucosa model	Human ACC cells and CAFs	CAFs promoted ACC cell invasion in 3D matrix in a spheroid fashion.	[43]

ACC, adenoid cystic carcinoma; CAFS, Carcinoma-associated fibroblasts; ECM, extracellular matrix; PLGA, poly(lactide-coglycolide); SEVs, small extracellular vesicles; TEGDMA, Triethylene glycol dimethacrylate.

**Table 2:** Salivary gland 3D culture models.

System	Method	Cell Type	Observations	References
Scaffold-based	Matrigel	Human submandibular SG stem/progenitor cells	The organoids treated with FGF-10 exhibited higher expression of salivary gland-specific markers and more sensitivity to neurotransmitters. Heterotopic transplantation of the combined cell spheres and mouse embryonic salivary gland mesenchyme. Limited clinical application.	[47]
Scaffold-based	Hyaluronic acid-based hydrogel	Human Parotid acini cells	Organized spheroids maintained over 100 days. Salivary biomarkers and maintained viability for over 3 weeks in vivo. Increased granule production and amylase staining.	[48]
Scaffold-based	Matrigel™-GFR + Alk inhibitors	Human SG cells	Organoid formation Induced swelling of the organoids by stimulation with carbachol. Long-term TNF-α stimulation suppressed AQP5 mRNA and protein expression.	[49]
Scaffold-based	Laminin-111 coated hydrogel + fetal mesenchyme + recombinant neurturin	Adult mice SG cells	Branching morphogenesis after the addition of neurturin.	[50]
Scaffold-based	Human Fibronectin and Human Basement Membrane Extract	Human salivary gland cells	Suitable for clinical use. Polarized acinar 3D units, expression of tight junction proteins, acinar proteins and acinar adhesion markers.	[51]
Scaffold-based	Natural extracellular matrix scaffolds recycled from human salivary tissue digests	Human salivary tissue digests	Method preserves the ‘residual connective tissue’ remaining after mechanical and enzymatic release of cells from human submandibular gland biopsies. Presence of collagen types I, III, and IV. Comparable fiber arrangement to original gland. Seeded epithelial cells and fibroblasts attached, proliferated, and were alive after 4–8 days of culture	[26]
Suspension culture	Serum-free scalable suspension culture	Human acinar and ductal cells	Partially digested human submandibular tissue. Cells attached to their native extracellular matrix components. Salivary spheroids called salivary functional units (SFU). Expressed acinar markers, polarization, and tight junction proteins	[57]
Microwells Culture	Micropatterned PEG hydrogel + PCL nanofibrous scaffold	Human parotid epithelial cells	Higher levels of salivary epithelial markers & tight junction proteins. Non-animal and serum-free culture system. Uniformed size spheroids.	[58]
Microwells culture	Micropatterned PEG hydrogel + PCL nanofibrous scaffold	Human single clonal SG stem cells	Higher levels of salivary epithelial markers& tight junction proteins. Higher α-amylase secretion and intracellular calcium levels. Two-stage niche independent culture system. Uniformed size spheroids	[59]
Bio-printing	Magnetized primary SG derived cells magnetic 3D levitation	Porcine primary SG-derived cells	Greater cell viability and pro-mitotic cells. Consistent organoid formation	[60]
Bio-printing	Gelled egg yolk plasma	Human salivary acinar cell line and primary fibroblasts	3D-printed gelled egg yolk plasma with controlled geometries. Manually extruded gelled egg yolk plasma bio-ink with fluorescence cells into a 3D-Cryo well insert and showed cell positioning.	[61]
Decellularized extracellular matrix-Hydrogel	SG tissue-derived decellularized extracellular matrix hydrogel	Rat SG stem/progenitor cells	Functional orthotropic bio-scaffold. Increased α-amylase level expression.	[63]

Alk, Activin receptor-like kinase; AQP, Aquaporin; FGF, Fibroblast growth factor; GFR, growth factor reduced; PCL; Polycaprolactone; PEG; Poly(ethylene glycol); SG, Salivary gland; TNF, Tumor necrosis factor.
